# Health tourism: an opportunity for sustainable development

**Published:** 2019-01-06

**Authors:** M Illario, V De Luca, L Leonardini, M Kucharczyk, AS Parent, C Dantas, AL Jegundo, W van Staalduinen, J Ganzarain, L Comisso, C Bramezza, AM Carriazo, A Maritati, G Tramontano, P Capozzi, E Goossens, C Cotrone, A Costantini, M Ciliberti, M Femiano, A d’Amore, M Forlenza, R Ruggiero, A Bianchi, L Augustin, V Marrazzo, T Dello Ioio, S Capaldo, A Crudeli, G De Cesare, F Cuccaro, G Bracale, D Tramontano, A Postiglione, C Matera, E Coscioni, J Bousquet

**Affiliations:** 1 Health Innovation Division of Campania Region (DG04), Federico II University and Hospital, Naples, Italy; 2 Research and Development Unit, Federico II University Hospital, Naples, Italy; 3 Programma Mattone Internazionale Salute, Italian Ministry of Health, San Donà di Piave VE, Italy; 4 AGE Platform Europe Network, Brussels, Belgium; 5 Caritas Coimbra, Coimbra, Portugal; 6 Academy on Age-friendly Environments BV, Gouda, the Netherlands; 7 Azienda per i Servizi Sanitari n.5 “Bassa Friulana”, Udine, Italy; 8 Azienda ULSS n. 4 Veneto Orientale, San Donà di Piave VE, Italy; 9 Junta de Andalucia, Seville, Spain; 10 Health Innovation Division of Campania Region (DG04), Federico II University and Hospital, Naples, Italy; 11 Center for Gastrology, Brussels, Belgium; 12 Relations with European and extra-European countries Division, Campania Region, Naples, Italy; 13 Azienda Sanitaria Locale Napoli 3 Sud, Castellamare di Stabia NA, Italy; 14 Azienda Sanitaria Locale Napoli 2 Nord, Frattamaggiore NA, Italy; 15 Azienda Sanitaria Locale Napoli 1 Centro, Naples, Italy; 16 Istituto Nazionale Tumori Pascale, Naples, Italy; 17 Regional Coordination of Tourism Districts of Campania, Naples, Italy; 18 Parco regionale dei Monti Lattari, Castellammare di Stabia NA, Italy; 19 Federalberghi Terme, Rome, Italy; 20 Federterme, Rome, Italy; 21 Centro Mediterranea Diagnostica Srl, Castellamare di Stabia NA, Italy; 22 Mediterranean Federation for Advancing Vascular Surgery, Naples, Italy; 23 Department of Molecular Medicine and Medical Biotechnology, Federico II University, Naples, Italy; 24 General Directorate for Health Protection and the coordination of Regional Health System, Campania Region, Naples, Italy; 25 Regional Ministry for Tourism, Campania Region, Naples, Italy; 26 Department of Heart Surgery, San Giovanni di Dio e Ruggi d’Aragona Hospital, Salerno, Italy; 27 Department of Pneumology and Addictology, Montpellier University Hospital Center, Montpellier, France

**Keywords:** Active ageing, health tourism, innovation, accessibility, age-friendly environments

## Abstract

In February 2017, the “Programma Mattone Internazionale Salute” (ProMis), that is the Italian Program for Internationalization of Regional Health Systems of the Ministry of Health (MoH), presented the first version of its Position Paper on Health Tourism, which embeds a first shared approach to the recommendations expressed by the European Committee of Regions (CoR) on “Age-Friendly” tourism. The CoR stresses the importance of local and regional authorities in the coordination of multi-sectoral policies such as healthcare, social assistance, transport, urban planning and rural development in relation to the promotion of mobility, security, accessibility of services, including health care and social services.

“Age-friendly” tourism is an example of an innovative tourist offer that strives to meet the health needs of the entire “traveling” population, with an integrated and cross-sector approach that involves various organizations operating in sectors such as healthcare, accessibility and transport.

The aim of the workshop was to explore the interest of the stakeholders to participate in a systemic action in the field of “health” tourism, and to identify priority implementation areas that offer opportunities to take advantage of validated, innovative experiences that strengthen the accessibility to health and social services in regional, national and international contexts.

This effort provides the opportunity to take advantage of aligning the European Structural and Investment Funds (ESIF) to the development of tourism, coherently with the needs and resources of local and regional health authorities.

## I. INTRODUCTION

Europe is the first tourist destination in the world, with the highest number and variety of destinations. The tourism industry is a key sector of the European economy, generating more than 10% of EU GDP and employing 9.7 million people and involving 1.8 million businesses. The 2007 International Convention on the Rights of Persons with Disabilities promotes “Tourism accessible to all”, promoting travel for the older adults and people with special access needs, as an integral part of any responsible and sustainable tourism policy. In the European tourism strategy the European Commission sets the objective of improving the accessibility of tourism services to sensitize the interested parties to generate greater know-how on the demand and profiles of travelers with specific needs and to evaluate the economic impact of the age-friendly tourism and patient-friendly [1]. Older adults represent a very important market share, but they also require an adaptation of services to take account of the specific needs. The same applies to the growing number of tourists with reduced mobility (recently estimated at 127 million people), people with disabilities or those suffering from chronic illnesses, whose needs must be integrated into the provision of tourism services [1]. Tourism for the older adults and people with special access needs presents difficulties with regard to social and health care, in particular due to the lack of integration between the health systems of the Member States and the tourism sector. This makes tourist destinations less accessible for people suffering from diseases.

## II. THE EMERGING MARKETS FOR HEALTH TOURISM IN THE SILVER ECONOMY

The workshop represents a follow-through of the satellite meeting held in Brussels on February 2018, to outline a collaborative work that to be carried out by ProMIS, the Reference Sites Collaborative Network (RSCN) of the European Innovation Partnership on Active and Healthy Ageing (EIP on AHA) [2–6], in the effort of transforming the challenge of an ageing population into a sustainable opportunity for development. The vision of the ProMis working group on Health Tourism, set up jointly by 9 Italian regions coordinated by Campania, that was shared with the RSCN of the EIP on AHA, has been laying the ground for a new model based upon a shared consensus [7].

Ageing is one of the greatest social and economic challenges facing the EU [8]. The ageing population includes several groups, with their own need-patterns: active, fragile and dependent, igniting age-specific spending priorities and patterns that are a part of the general consumer economy, and are identified as “Silver Economy”. The Silver Economy is driven by the emergence of a new consumer market and by the need to improve the sustainability of public expenditure linked to ageing, as goods and services for active and healthy ageing are likely to impact on the efficiency of healthcare and social security systems, increasing their sustainability [9].

The tourism sector provides opportunities arising from the improvement of health services to residents, that could strengthen a personalized service offer targeting tourists as well. This could be exploited to increase attractiveness of internal areas as well as of out of season offers, more sustainably by including activities that positively impact health. Indeed, the turnover generated by health tourism is around 2 billion Euro, and could further grow [10].

The European directive 2011/24/EU on cross-border healthcare [11] represents a further growth opportunity for European health system and an extraordinary vehicle to strengthen the ties between the commercial enterprises and the health sector of the Member States (MS). According to Directive, healthcare providers should make available relevant information to help individual patients to make an informed choice, including treatment options, cross-border service availability, quality and safety of healthcare provided in the MS of destination. The efforts of the stakeholders are being channelled into platforms, networks and partnership to influence policy makers and accelerate the action towards implementation of innovative approaches. Age-Platform Europe is a non-governmental organization (NGO) representing older adults at the EU level, inspired by the will of older people to become considered in the elaboration of an “age-friendly” market offer for the different social-economic context. Silver economy, silver tourism and active and healthy ageing are its key-words [9].

The diversity of older people goes across the different age-ranges that express diverse needs in terms of accessibility of services and socioeconomic backgrounds, physical impairments, illnesses, disabilities, gender differences, loneliness, social exclusion.

Healthy life years versus overall life expectancy can be translated into services for older adults, as well as the need to support mobility, and services that are health and well-being targeted.

People aged 50+ spend a quite large amount of money in tourism, and probably health tourism is a “niche” market, where also age-friendly tourism should look at.

Silver tourists like to travel with companions, extend the visits to family and friends, are not interested in “all inclusive” packages, like wellness, recreation and health related offer (rheumatism, balneotherapy, dermatology, etc.), gastronomic tour. They are interested in history and commemorations.

Interestingly, they prefer autumn and spring rather than high season, when longer stays can mitigate the feeling of loneliness and isolation, especially where the offer for health services is provided and represents an added value. Older adults can be tourists, but also guests: becoming actively involved in the set-up of a personalized offer for tourism among peers might become a new approach to set up the tourist offer.

An important focus is on accessibility although it is not only about built infrastructure but also about transfer and access to health services.

People use more and more ICT devices especially when they travel, hence need to adapt new technologies for the needs of older people.

Since the physical and psychological vulnerability, real or perceived, of older people, there is the need to ensure a feeling of safety by strengthening service offer across the domains that directly or indirectly impact health and wellbeing. Going beyond to improve the tourism offer needs to focus on social goals of tourism, also taking into account people with lower income, with disabilities, and opportunities arising in remote areas. Disease prevention and health promotion applied to active ageing can be adapted to specific needs: ex. Sports, physical activity, food and nutrition with a life-course approach. Integrated care also can improve the service offer, coupled with strategies and initiatives for AHA at the national and regional levels to facilitate service provision to residents and tourists. In this perspective, the engagement of municipalities is key to ensure availability of social services as well as multi-organization commitment to ignite the involvement in this new sector of the tourism market [12–13].

## III. ADDRESSING THE GAPS ON “SMART HEALTHY AGE-FRIENDLY ENVIRONMENTS” (SHAFE)

SHAFE is a cross-sectoral Thematic Network involving health, social care, the building industry and the ICT industry, coordinated by Caritas Coimbra and AFEdemy, with over 120 organizations with thousands of stakeholders. It was built on the work of the EIP on AHA and was approved by the European Commission for 2018. The network was launched to focus on aligning technological development with the building industry for smart environments in terms of policy and funding, enhancing a more efficient health care system that may add better quality for less investments.

The specific goal of SHAFE was to provide recommendations that may improve the quality of life of older adults and the places in which they live through the effective implementation and broad adoption of innovative and sustainable eHealth and mHealth solutions. This work focused mainly on intelligent environments to support patients suffering from chronic diseases and impairments - through the use of robotics, domotics and intelligent communications with formal and informal care [14].

The Network also provided good practices that blend health and care services, connecting tools and building environment. Cities and rural environments are addressed, to allow people to age well without any constraints [15]. SHAFE highlights that age-friendly tourism requires an integrated and cross-sector approach, involving many different stakeholders. Most facilities are not equipped for people with specific needs for health: hence the need for coherent investments to improve accessibility to tailored service provision.

## IV. TRANSFERABLE ELEMENTS EMERGING FROM THE GOOD PRACTICES IN HEALTH TOURISM

The HoNCAB is European project addressing cross-border healthcare.

One of the specific objectives of the project [16], was to investigate existing experiences of cross-border care, with a focus on “direct” cross-border healthcare and “health tourism”. The project highlighted some transferable elements to health tourism, and the need for new regulation to support care service provision that is digitally supported, to:

- Patients traveling purposely to receive specific health care services;
- Patients traveling for tourism;
- Emergency services.

Another example of good practice was the pilot experience of social and inclusive tourism coordinated by the ULSS 4 Eastern Veneto, aimed at tourists with special needs that involved 10 municipalities in Veneto coast. The project made available several sport activities for disabled people. This has been possible thanks to the organization provided by the management of the local health agency, strongly endorsed by Veneto Region.

Montpellier and surrounding municipalities also validated a good practice to screen for fall prevention initiative available at Balneotherapy centers [17]. This intervention study foresaw balneotherapy/thermal cure embedding a fall prevention exercise approach on 1471 patients.

The Gastrological Approach to Malnutrition represents a primary nutritional approach to prevent and treat malnutrition in the older adults and in patients affected by diseases that alter the perception of taste, based on an integrated vision developed by “Food and Nutrition” Action Area of the Action Group on “Lifespan Health Promotion and prevention of age-related Frailty and Disease” (A3 AG) of the EIP on AHA [18–20]. The Center for Gastrology (Leuven, Belgium) developed a tool to profile individual taste disturbances in order to prescribe recipes to personalize meals for cancer patients. Through an IT platform, a multidisciplinary team of doctors, nutritionists and cooks interfaces with the patient to realize a personalized nutritional intervention, which considers the specificity of the disease, safeguarding the taste and quality of food. The gastrological approach has an cultural embeddedness, therefore this finding opens up promising perspectives in the context of health tourism.

The “Pizza Pascalina” exploits the communication power of food to disseminate a message of healthy lifestyles. Its Mediterranean style recipe includes products that are rich in anti-oxidants and carotenoid that reduce proliferation of cancer cells in “in vitro” studies, as well as extra-virgin olive oil that is rich in polyphenols, that are protective against cardiovascular risk and cancer, and crucifer vegetables that are rich in sulphur agents. Although many studies demonstrated the positive effects on health of the Mediterranean Diet (MD), the adherence to this food regimen is still too low, reducing the health gain related to MD [21].

The MD has been recognized by UNESCO as an Intangible Heritage of Humanity in 2010. In addition in 2012 the MD has been included by the FAO at the top of the list of the most sustainable diets in the planet [22]. The double recognition of healthy diet and life style generated a new approach to this cultural heritage by the stakeholders who are progressively recognizing that it may become a new tool to develop green economy and sustainable tourism.

MD relates to the territorial development based on immaterial assets such as the emblematic communities. The aim of the MD.net Interreg MED project is to identify routes and activities of international cooperation aimed at stimulating local development of the MD emblematic communities. The focus of Campania in this project is the Cilento area.

## V. PROJECTS BLENDING AND FUNDS COMPLEMENTATION TO SUPPORT HEALTH SERVICES

Our health system asks for changes in the way healthcare is delivered, organized and financed across the Europe. Health authorities are looking for ways to put more emphasis on prevention and shift activities from hospitals to primary and community, integrating services across the levels of care and between social and healthcare. These efforts focus on people needs and take advantage of the potential of digital technologies.

In order to deliver the expected benefits, two very important preconditions are needed:

- sufficient mobilisation of investment,
- capacity to design and implement the new care models.

The fundamental elements to engage in such investment mobilisation are:

1. Long term investment strategies, integrating infrastructure, innovative technologies and new care models;
2. Integrated investment strategy requires to blend financing from different sources;
3. Investments blending requires the development of partnerships with new stakeholders, new investors, implementing a different way to manage the new financing instruments.
4. Contracting and payment models need to be considered in conjunction with the planned investment, to assess efficacy.

DG Sante, has been carrying “European Structured Funds for Health” initiative to map all the structured fund actions of National and regional authorities that have been supporting health in the current financial framework, showing the diversity and the creativity of the projects to be supported by the structured funds.

Next Multiannual Financial Framework (MFF) will start after 2021, when the structural funds will continue very strongly to be an opportunity for those that want to invest in health and in what is important for health: it is pivotal for regions to engage in the process of programming as early as now.

A number of support instruments in form of loans or instruments for investors (European funds for strategic investments - EFSI) is going to continue being an opportunity in the next multiannual financial framework.

The reform process paralleled by the right knowledge on how to implement new models of care is needed to make sure that the implementation can be successful. Therefore it is important to develop technical support and capacity building for regional authorities, through networking between authorities through the EU Health programme. In the next MFF, projects will be financed to identify the transfer and replication of best practices to new locations and in particular those addressing chronic disease.

A strategic approach should be developed to make the best use of the digital transformation of health and care. In recent policy paper [23], specific actions from the EU/national level have been identified to facilitate the wide use of digital tools for citizens and patients centred care.

## VI. THE CHALLENGES OF WELLBEING AND TOURISM FOR HEALTH SYSTEMS

Health tourism is an opportunity of sustainable development that can be declined along several directories, taking advantage in Europe of the cross-border availability of services for health. Sharing data and allowing data retrieval for specific citizens is an important enabling factor to the set-up of personalized offer for health to strengthen the tourist offer.

There is clearly a need to profile the “tourist clients” in order to set up adequate offers and business models, implementing the activities for example of thermal stations for health promotion and disease prevention while strengthening their use for residents, to open to tourists as well.

Enabler to the exploitation of the health tourism market is the need to balance accessibility and equity, and to be careful in maintaining the communication with the patient/tourist to support inclusive tourism.

There have been several initiatives trying to support value-based collaborations among Mediterranean countries/regions, highlighting the potential role of Campania as a coordinator in the framework of Mediterranean collaboration.

The Mediterranean Federation for Advancing Vascular Surgery (MeFAVS) aims to promote and strengthen the cooperation between the reference organizations involved in Vascular Surgery and the countries of the Mediterranean basin: in particular Italy, France, Spain, Greece, Portugal, the Balkan area, Lebanon, the Middle East, the United Arab Emirates, Egypt, Morocco, Tunisia and other countries that face/are close to the Mediterranean. MeFAVS addresses the need of sharing the know-how in vascular surgery and build bridges among health systems, working jointly to bridge the differences and foster the progress of vascular surgery throughout the Mediterranean area.

There is an urging need of framing the service provision for health to the different traveling populations (tourists, patients migration, wellness, etc.), highlighting whenever possible the offer of new, special services.

An important directory develops along the valorisation of the offer deriving from the networking between thermal locations. It is very important to identify the need for services expressed by the tourist demand, strengthening the offer according to the evolving changes depending on the tourist fluxes. This translates for service providers in the mobilization of an incoming economic flux that also impacts beyond the health system. The development of policies for health tourism are multi-sectoral, where health services are an enabler. Strengthening the offer directed towards special health needs also in terms of health promotion is the key to take advantage of the opportunity for sustainable development of health tourism.

Safety, quality, accessibility of services for baby-boomers and older adults can be achieved by a structured public/private network that is capable of providing services along the emergency, chronicity and wellbeing.

It is pivotal to design and implement a targeted communication plan on the services that are available, and how they can be accessed, according to a sustainable business model. The need for a strengths and weaknesses assessment is urgent to identify the priority actions to undertake.

## VII. OPPORTUNITIES FROM THE INDUSTRIAL SECTOR

The trends for health tourism in Italy are in constant growth, thanks to a number of initiatives that valorise the different offers. The industrial sector expressed the need for greater clarity in administrative processes that can stimulate sustainable development and investments. The approach towards administrative simplification of Campania region for touristic activities takes advantage of a “silent-assent” rule, aimed at increasing attractiveness for investors. Often the wealth of the territories suffers from lack of coordination, networking and adequate communication activities that impact on the use of local resources. Campania Touristic Districts are aimed at creating a de-centralized offer for tourism. They are moving in the field of communication, and are exploring intercontinental avenues where it is possible to move as a cluster and not in a fragmented way.

The viewpoint of the industrial sector focuses on the objective to strengthen the attractiveness of the Italian health sector. In the specific case of the thermal sector it is more immediate to identify the specific fields of application for specific target populations, eg. fall prevention initiatives. Prevention is not financed by the Health System, nonetheless it could be possible to re-invest part of the avoided cost in health tourism. The thermal services focus on the tourism for wellbeing and prevention of diseases, through a number of interventions that span from health promotion to cure. It is important to identify a shared strategy for the sector in order to compete at the international level for tourist influxes, such as the setup of an European Grouping of Territorial Cooperation (EGTC).

## VIII. CONCLUSIONS AND REMARKS

The health care system should no longer be regarded as a cost-generating sector, but rather as a driving force for the economy. Quality health benefits and services can attract citizens who are concerned about maintaining health and well-being as well as patients in search of high-quality services, thus contributing to the development of the tourism market. In this framework, the phenomenon of health tourism is promising as a global positioning of the tourist sector in Italy and in the EU, for sustainable economic development.

There is a need to define a normative regulation, which supports the development of health tourism, in synergy with the other sectors that are directly or indirectly involved in the effort of strengthening the attractiveness of the entire national territory, taking into account the ongoing digital transformation of health and care.

Health tourism can have a tangible development: in this sense it is necessary that the world of health and tourism sit together to agree on the development of a common planning, that takes into account the strengths and peculiarities of each sector, in a synergic and virtuous perspective. It is therefore essential that both sectors share experiences and integrate each other’s excellence while maintaining the focus on citizens and on the demographic evolution. The aim of such a new model should allow both the maintenance of quality and sustainability of health and social services, as well as stimulating the tourism attractiveness of proposing areas, integrating digitally supported well-being and health promotion services, encouraging active and healthy ageing.
